# Outpatient Primary Care Practitioner Access: Gender-Based Preferences

**DOI:** 10.1089/whr.2021.0088

**Published:** 2022-02-02

**Authors:** Saira J. Khan, Kenneth G. Poole, Juliana M. Kling, Gretchen Taylor

**Affiliations:** ^1^ Internal Medicine Residency, Mayo Clinic Arizona, Scottsdale, Arizona, USA.; ^2^ Office for Provider Advancement, Optum Care, Eden Prairie, Minnesota, USA.; ^3^ Division of Women's Health Internal Medicine, Mayo Clinic Arizona, Scottsdale, Arizona, USA.; ^4^ Center for Women's Health, Mayo Clinic, Rochester, Minnesota, USA.; ^5^ Division of Internal Medicine, Mayo Clinic Arizona, Phoenix, Arizona, USA.

**Keywords:** primary care, patient-centered care, access, patient experience, gender

## Abstract

***Background:*** Primary care practices are evolving under the pressure of modern-day challenges, with some clinics now introducing the choice of new nontraditional care models designed to maximize patients' needs with practitioner efficiency. These changes include team models consisting of advanced practitioners and physicians, as well as new care delivery formats such as virtual care. With a growing number of options for care, it is unclear whether patients' gender affects their visit preferences; therefore, we surveyed patients presenting to an outpatient internal medicine clinic in Arizona to understand how practice variations impact patient satisfaction of their primary care.

***Methods:*** Patients seen in an outpatient internal medicine clinic were surveyed. Multivariable models adjusting for age, marital status, education level, and income were used to evaluate gender-based care preferences.

***Results:*** Of 796 total participants (446 women, 350 men), women were more likely to prefer continuity of care with the same health care practitioner (90.2% women vs. 85.0% men, *p* = 0.028) and allied health staff (AHS) (36.3% women vs. 28.0% men, *p* = 0.0031) over convenience of appointment or quicker response time than men. However, after multivariable analysis, no statistically significant relationships remained.

***Discussion:*** Women favored both continuity of care with the same health care provider and AHS over faster access to primary care. A large majority of men had similar preferences for continuity of care. To provide the highest level of care with greatest patient satisfaction, understanding individual preferences for care delivery will be important.

## Introduction

Continuity of care has long been recognized as a fundamental aspect of the current outpatient primary care model.^1^ However, as a shortage of primary care physicians faces an aging patient population with rising complexity of chronic diseases, the primary care system has been challenged to maximize patient volumes with practitioner efficiency to meet increasing demands.^1^ As a result, new nontraditional models of care are being explored.

From team-based care, where more than one practitioner works collaboratively on patient care, to virtual care, where care can be given despite long distances, wait times to see a practitioner can be drastically decreased that may lead to increased patient satisfaction.^2^ However, when it comes to maximizing satisfaction, patients are faced with the trade-off: continuity of care or quick access to any health care practitioner (HCP).

Various studies have sought to identify the factors that influence these health care seeking behaviors; determinants such as sex, age, and access to care have been shown to affect patient's health care utilization, but findings have been overall inconsistent.^3^ Furthermore, these studies largely have not compared use by preference, only by consumption. Differentiating utilization from preference is key in understanding patient priorities; for example, although multiple studies have found that while per capita health care costs—and by comparison, utilization—are 20%–30% higher for women than men, adjusting for sex-specific conditions, such as reproduction, almost completely negated this cost of use difference.^4^

To better understand health care utilization as a reflection of behavior, we examined gender-based differences in preference of outpatient primary care models. Identifying determinants behind health care seeking behavior and how they may differ between women and men could help tailor health care delivery to ultimately improve overall health outcomes.

## Methods

A 13-question survey was offered to all patients seen in an outpatient internal medicine clinic at Mayo Clinic Arizona for a 6-week period from January 2018 through March 2018. No patient identifiers were obtained. Because the institutional review board (IRB) determined the study to be IRB exempt as an anonymous survey, formal consent was not obtained from respondents. No exclusion criterion was utilized, and the survey was provided in English only. The patients returned the surveys in anonymous collection boxes at the nursing station outside the examination rooms upon completion.

The Mayo Clinic provides longitudinal internal medicine primary care in the greater metropolitan Phoenix area. Within the division surveyed, there are ∼25 practitioners, including several nurse practitioners (NPs) and physician assistants (PAs) who are assigned to teams of seven or eight practitioners each. When patients are unable to see their own practitioner, they are typically seen by someone within the same team.

The questionnaire included four hypothetical scenarios, using discrete choice experiment methodology, to assess respective patient preferences. These included (1) annual physical and/or routine follow-up; (2) acute illness or new health concern; (3) virtual visits, such as using video, versus face-to-face; (4) communication with allied health staff (AHS) such as nurses and medical assistants.

Respondent demographics were included in the survey, including age, gender, level of education, average income, age of children, length of time as a patient, and type of practitioner seen (physician vs. PA or NP).

Descriptive statistics and chi-squared tests were used to evaluate survey results using gender as a categorical variable. A Mann–Whitney rank-sum test was used for continuous variables. Multivariable models were adjusted for age, marital status, education, and income. *p*-Values <0.05 were considered statistically significant.

## Results

A total of 796 of 1731 (response rate of 46%) patients completed the questionnaire. Of the respondents, the majority identified as women (56%) and over the age of 65 years (58%) without a child under 18 years at home (92%). Only one participant (≤0.01%) declined response to gender identification, answering “prefer not to answer.” No participants responded with a write-in answer regarding gender identification. Greater than 60% of respondents reported an annual household income of >$100,000, with nearly all reporting their highest education level as “some college or associate degree” or higher. At least two-thirds (66%) of the respondents had been a Mayo Clinic patient for >5 years (Table 1).

**Table 1.** Survey Respondent Demographics

	Total (%)
Patient gender	796
Female	446 (56)
Male	350 (44)
“Prefer not to answer”	1 (<0.01%)
Write-in response	0 (0.00%)
Age (years)	796
<35	31 (4)
35–50	78 (10)
51–64	222 (28)
≥65	466 (58)
Annual household income	563
<$50,000	54 (10)
$50,000–$99,000	123 (22)
$100,000–$250,000	229 (40)
>$250,000	157 (28)
Child under age 18 years at home	563
Yes	61 (8)
No	681 (92)
Highest attained education	722
High school	57 (8)
Some college or associate degree	176 (24)
Bachelor's degree	235 (33)
Master's degree	146 (20)
Professional degree	59 (8)
Doctoral degree	49 (7)
Years at [clinic name]	792
<1	104 (13)
1–5	163 (21)
>5	525 (66)

When evaluating gender-based preferences, women were more likely than men to prefer communicating with the same AHS over receiving a faster response (36.3% women vs. 28.0% men, *p* = 0.0031) (Table 2). Women more often prioritized seeing their own HCP as opposed to scheduling a convenient appointment for routine care (90.2% women vs. 85.0% men, *p* = 0.028).

**Table 2.** Preferences by Gender

	Women (***n*** = 446)	Men (***n*** = 350)	Total (***n*** = 796)	***p***-value
When visiting the clinic for yearly physicals and/or routine medical follow-up				0.02801
Seeing my provider	369 (90.2%)	273 (85.0%)	642 (87.9%)	
No preference	19 (4.6%)	31 (9.7%)	50 (6.8%)	
Getting an appointment when I want it	21 (5.1%)	17 (5.3%)	38 (5.2%)	
When you are sick (for example, with the flu), or have a new health concern				0.10721
Seeing my provider	178 (44.5%)	123 (38.3%)	301 (41.7%)	
No preference	58 (14.5%)	63 (19.6%)	121 (16.8%)	
Getting an appointment when I want it	164 (41.0%)	135 (42.1%)	299 (41.5%)	
Virtual visits versus face-to-face visit				0.50991
In person	255 (62.2%)	216 (66.3%)	471 (64.0%)	
No preference	74 (18.0%)	54 (16.6%)	128 (17.4%)	
Getting a virtual visit with any provider when I want it	81 (19.8%)	56 (17.2%)	137 (18.6%)	
Communication with nurses and medical assistants				0.00311
Same nurse or medical assistant	147 (36.3%)	92 (28.0%)	239 (32.6%)	
No preference	61 (15.1%)	79 (24.1%)	140 (19.1%)	
Receiving a quick response	197 (48.6%)	157 (47.9%)	354 (48.3%)	

In the event of an acute illness, respondents showed no clear preference of seeing the same practitioner versus quick appointment time (*p* = 0.1072). Overall, in-person appointments were preferred over video, and no statistically significant differences in gender-based preferences was seen for which practitioner they saw by video (Table 2). In multivariable models adjusted for age, marital status, education level, and income, no statistically significant findings were identified (Table 3).

**Table 3.** Preferences Adjusted for Age, Marital Status, Education Level, and Income

Preference	Events/total (%)	Odds ratio	***p***-value
Virtual visit			
	105/434 (24.2)	(95% CI)	
Women versus men		1.13 (0.70–1.81)	0.61431
Getting acute care appointment when you want it			
	223/416 (53.6)	(95% CI)	
Women versus men		1.03 (0.68–1.56)	0.89821
Getting routine annual care appointment when you want it			
	35/619 (5.7)	(95% CI)	
Women versus men		0.71 (0.34–1.44)	0.33861
Communication with same AHS			
	161/431 (37.4)	(95% CI)	
Women versus men		0.94 (0.62–1.44)	0.79181

AHS, allied health staff.

## Discussion

Predominantly white well-educated women presenting to an outpatient clinic in Phoenix, Arizona, not only preferred continuity of care with the same HCP, but also preferred AHS consistency over quick response time or appointment time. This finding is consistent with data published in 2018 by Liu et al. who also found that female patients have stronger preference for seeing their own doctor in an act of risk aversion; for example, by maintaining continuity of care, they have found those who utilized continuity of care with the same HCP have been found to have both decreased emergency department use and hospitalization rates.^5,6^

More broadly, our study revealed that men were largely in favor of continuity of care as well, unlike the study by Liu et al., which found that males prefer shorter appointment delays over returning to the same HCP.^5^ This may be related to the multicomorbid and highly complex patients who seek care at our clinic as a tertiary center. Overall, health comorbidities seem to matter. Even when patients were experiencing virtual visits for the first time, those with three or more complex health conditions have been found to be more likely to have seen that same HCP before the virtual visit.^7^

Regarding preferences around acute visits, or visits for a new medical condition or illness, no statistically significant difference was found between gender-based preferences when accounting for age, marital status, years of education, and income. Therefore, when designing acute care models, continuity of HCP may not need as of strong an emphasis when compared with long-term care.

After adjusting for multiple variables that may influence the identified gender-based preferences for continuity of care, these relationships were no longer found to be significant. This may indicate that other factors play a larger role in preference determination. Age, for example, has been shown to affect health care utilization behaviors; McGrail et al. found that patients aged 20–44 years used 1.5 to 6 times as many virtual visits as other age groups.^7^

It is also possible that because the majority of the patients in our survey were >50 years old, and prior studies have shown that older age cohorts attend up to 46% more primary care visits than their younger counterparts, there is not yet sufficient data to fully evaluate preference trends in these younger cohorts.^8^

Marital status, too, may affect these gender-based health care preferences. A substantial amount of literature supports that marriage has a positive effect overall on health, including a longer life expectancy, improved mental health, and overall better satisfaction with quality of life.^9–12^ Perhaps the overall better health among married men and women and, therefore, potentially lower complexity needs, allows for more flexibility in obtaining primary care; therefore, the necessity of continuity of care may be diminished in married individuals compared with their single counterparts.

That said, due to its recent national legalization in 2015, the impact of same-sex marriage on health and behavior, versus heterosexual marriage, remains unclear. For example, although the California Health Interview Study found that same-sex legally partnered/married respondents were more likely to have health insurance and use health care more than their single counterparts, lesbian couples, when compared with heterosexual counterparts, were significantly less likely to report good or excellent health.^13^

Furthermore, lesbian partnerships reported less use of a consistent source of care, which should be taken into consideration when addressing gender-based preferences.^13^ Additional study is needed to better understand the potential influence of same-sex relationships with regard to health care delivery to individualize their experience in health care.

The strength of this study includes its response rate, and representation of both men and women for adequate comparison. However, this study also has limitations. As a single-center study, surveyed patients were largely of a homogeneous population. Respondents were able to choose between “female” or “male” identification, could elect to not answer, or write in their own gender identification. Of the respondents, only one elected to not disclose gender identification, and the corresponding survey response was otherwise incomplete.

In addition, the survey was only administered in English, further limiting study participants; this may have also produced misunderstanding in patients for whom English is not a primary language. These English-only surveys were chosen based on the largely English-speaking population of the Mayo Clinic, and it is unlikely that providing a second language would have provided significant difference.

Further patient demographic questions, such as ethnicity, insurance type (commercial vs. Medicare/Medicaid), and number of common comorbidities, were not obtained in this survey. This information would help further stratify the study's findings. In addition, some surveys had unanswered demographic questions, and others had to be discarded due to uninterpretable responses. Lastly, this study was conducted before the novel coronavirus (COVID-19) pandemic, which necessitated the use of virtual care that may have been met with hesitation before the pandemic. Therefore, it would be advantageous to replicate this study, especially as it relates to virtual care, to see whether the pandemic experience has modified any of the identified gender-based relationships.

We cannot exclude the possibility that some respondents may have completed the survey more than once. To ease distribution of this paper survey, and to ensure complete anonymity, respondents were not tracked. However, because very few patients are seen multiple times in a 6-week period through the Mayo Clinic, the likelihood of this occurring is quite low.

Despite its limitations, this study provides insight into how outpatient care models can adapt to diverse patient care preferences—as demonstrated by clear differences in gender-based preferences. Overall, women favored longitudinal care with the same HCP and AHS, signifying that they would be willing to sacrifice conveniences such as shorter appointment delays or staff response times. Providing various care models to patients within a single practice, including the ability to maintain consistency with a single HCP, would seem the best approach to optimizing patient satisfaction. Otherwise, aiming to increase the use of continuity of care for women may lead to higher patient satisfaction in practices that deliver high volumes of annual routine medical care.

## Conclusion

Gender-based differences may exist in patient preferences regarding primary care access. In Caucasian well-educated women, continuity of care with the same HCP and AHS was preferred over ease of scheduling or quick response time, but this preference may be explained by other factors such as age or marital status. However, a majority of the surveyed men revealed similar preferences in their primary care. Future evaluation of these relationships longitudinally and inclusive of other demographics, such as socioeconomic factors, would allow for an individualized and inclusive experience for all in this era of evolving practice models.

## Abbreviations Used

AHS: allied health staff
COVID-19: coronavirus disease-2019
HCP: health care provider
NP: nurse practitioner
PA: physician assistant
